# Asthma–Chronic Obstructive Pulmonary Disease Overlap Syndrome Associated with Risk of Pulmonary Embolism 

**DOI:** 10.1371/journal.pone.0162483

**Published:** 2016-09-09

**Authors:** Jun-Jun Yeh, Yu-Chiao Wang, Chia-Hung Kao

**Affiliations:** 1 Ditmanson Medical Foundation Chia-Yi Christian Hospital, Chiayi, Taiwan; 2 Chia Nan University of Pharmacy and Science, Tainan, Taiwan; 3 Meiho University, Pingtung, Taiwan; 4 Management Office for Health Data, China Medical University Hospital, Taichung, Taiwan; 5 College of Medicine, China Medical University, Taichung, Taiwan; 6 Graduate Institute of Clinical Medical Science, College of Medicine, China Medical University, Taichung, Taiwan; 7 Department of Nuclear Medicine and PET Center, China Medical University Hospital, Taichung, Taiwan; 8 Department of Bioinformatics and Medical Engineering, Asia University, Taichung, Taiwan; Lee Kong Chian School of Medicine, SINGAPORE

## Abstract

**Purpose:**

We conducted a cohort study to clarify this relationship between asthma–chronic obstructive pulmonary disease (COPD) overlap syndrome (ACOS) and pulmonary embolism (PE).

**Methods:**

From the National Health Insurance Research Database of Taiwan, we identified patients who had a diagnosis of asthma and a diagnosis of COPD (defined as ACOS) and concurrent treatment between January 1999 and December 2009 (ACOS cohort: *n* = 14,150; non-ACOS cohort: *n* = 55,876). Cox proportional hazards regression analysis was performed to determine the adjusted hazard ratios (aHRs) for PE of the ACOS cohort compared with the non-ACOS cohort.

**Results:**

Comparing the ACOS cohort with the non-ACOS cohort, the aHR of PE was 2.08 (95% confidence intervals [CIs]: 1.56–2.76). The risk of PE was higher in ACOS cohort than non-ACOS cohort, regardless of age, sex, comorbidity, inhaled corticosteroids (ICSs) and oral steroids (OSs) used. For ages ranging from 20 to 65 years, the aHR of PE was 2.53 (95% CI: 1.44–4.44) in the ACOS cohort. ACOS patients using ICSs (aHR: 1.97, 95% CI: 1.29–3.01) or OSs (aHR: 1.97, 95% CI: 1.46–2.65), the risk of PE was higher than in the non-ACOS cohort. The risk of PE increased with the number of outpatient visits and hospitalizations necessitated, ranging from 2.32 (95% CI: 1.54–3.52) in patients having 3–9 visits to 4.20 (95% CI: 2.74–6.44) for those having >9 visits.

**Conclusions:**

ACOS is associated with increased risk of PE, particularly patients with a high frequency of AE—even in young adults or people without comorbidities.

## Introduction

Pulmonary embolism (PE) is a life-threatening disease [1], the incidence of which has been increasing according to a recent report [2]. High mortality is associated with a delayed diagnosis of acute PE [3]. Recent studies have indicated that the predisposing factors for PE include numerous underlying diseases such as obstructive airway disease (causing asthma [4] and chronic obstructive pulmonary disease [COPD] [5]), atherosclerosis [6] (causing hypertension, diabetes [7], and stroke), heart failure [8] and cancer [9].

The triad of abnormalities in hypercoagulability (blood composition), endothelial injury (vessel wall function), and stasis (blood flow) has been attributed to the shear stress involved in venous thrombosis or thromboembolism [10]. In addition, the airways represent a body compartment in which coagulation self-initiates [11]. hypoxeamia -induced vascular endothelial dysfunction [12] reduces the activation of endothelial nitric oxide in patients with obstructive airway disorders [13]. Therefore, the levels of endogenous thrombin potential (ETP), plasminogen activator inhibitor type 1, and von Willebrand factor (vWF) increase [14]; primary pulmonary vascular remodelling [12] and increased vasoconstrictor tone may contribute to in situ thrombosis [4,5]. This combination of factors in acute exacerbation (AE) in asthma and COPD primarily results in PE without deep venous thrombosis [15,16]; this supports the notion that asthma and COPD with hypoxeamia are critical factors of PE [17].

Asthma-COPD overlap syndrome (ACOS) is a disorder comprising both asthma and COPD. The infiltration of neutrophil and eosinophil [18] in the airway caused by ACOS [19] are similar to the pathophysiology change of the airway in severe asthma [20] and COPD [21,22]. According to Ghebre et al., neutrophil plays a key role in ACOS [22]. Moreover, the level of neutrophil cells correlates with the levels of ETP and vWF, which play key roles in severe asthma with PE [14]. No previous report has mentioned the association between PE and ACOS. We hypothesized that ACOS is associated with the risk of PE; accordingly, we conducted a cohort study to evaluate this association.

## Methods

### Data Source

The study population cohort was established using data from the Longitudinal Health Insurance Database 2000 (LHID 2000), a subset of the National Health Insurance Research Database (NHIRD) of Taiwan. The NHIRD is a database of all registry and claims data from the National Health Insurance (NHI) program, including data on patient demographic characteristics, diagnoses, and prescription claims for ambulatory and inpatient care. The NHI program is a mandatory single-payer national health insurance scheme that has provided comprehensive medical care coverage in Taiwan since 1995, covering more than 99% of all residents in Taiwan. The LHID 2000 data set consists of claims data collected from one million people randomly selected from the total population of insurants in the 1996–2011 period. The International Classification of Diseases, Ninth Revision, Clinical Modification (ICD-9-CM) is used to record diseases in the database. According to NHIRD procedures, the original identification numbers for insurant files were replaced with anonymous numbers for privacy protection.

### Ethics Statement

The NHIRD encrypts patient personal information to protect privacy and provides researchers with anonymous identification numbers associated with relevant claims information, including sex, date of birth, medical services received, and prescriptions. Therefore, patient consent is not required to access the NHIRD. This study was approved to fulfill the condition for exemption by the Institutional Review Board (IRB) of China Medical University (CMUH104-REC2-115). The IRB also specifically waived the consent requirement.

### Data Availability Statement

All data and related metadata were deposited in an appropriate public repository. The data on the study population that were obtained from the NHIRD (http://nhird.nhri.org.tw/en/index.html) are maintained in the NHIRD (http://nhird.nhri.org.tw/). The NHRI is a nonprofit foundation established by the government. Only citizens of the Republic of China who fulfill the requirements of conducting research projects are eligible to apply for the NHIRD. The use of NHIRD is limited to research purposes only. Applicants must follow the Computer-Processed Personal Data Protection Law (http://www.winklerpartners.com/?p=987) and related regulations of National Health Insurance Administration and NHRI, and an agreement must be signed by the applicant and his/her supervisor upon application submission. All applications are reviewed for approval of data release.

### Study Population

Employing a retrospective population-based cohort study design, we investigated the association between asthma–chronic obstructive pulmonary disease overlap syndrome (ACOS) and PE outcome. ACOS patients were defined as COPD (ICD-9-CM Codes: 491, 492, and 496) patients aged >20 years with concurrent [23] physician-diagnosed asthma (ICD-9-CM Code: 493) [24,25,26]. The index date represents the date of asthma diagnosis. We collected data for 14,150 ACOS patients who received treatment between 1999 and 2009 to establish the ACOS cohort. For each ACOS patient, four comparison subjects were matched for sex, age (5-year intervals), and index year; these subjects comprised patients not conforming to the diagnosis condition of ACOS (non-ACOS cohort; *n* = 55,876). Diagnosis of PE (ICD-9-CM: 415.1) during the follow-up period represented the outcome variables. Subjects with a history of PE at baseline or those aged<20 years were excluded.

Follow-up person-years were calculated for each subject from index date until December 31, 2011, through to the date of diagnosis of PE or withdrawal from the insurance system. Preexisting comorbidities for all subjects comprised atrial fibrillation (ICD-9-CM: 427.31), hypertension (ICD-9-CM: 401–405), diabetes (ICD-9-CM: 250), hyperlipidemia (ICD-9-CM: 272), stroke (ICD-9-CM: 430–438), heart failure (ICD-9-CM: 428), lower limb fracture (ICD-9-CM: 820–823), and cancer (ICD-9-CM: 140–208). Use of inhaled corticosteroids (ICSs) or oral steroids (OSs) [27] was measured until the study end date. The ICSs were included beclometasone (ATC code: R03BA01), budesonide (ATC code: R03BA02), fluticasone (ATC code: R03BA05), ciclesonide (ATC code: R03BA08), and formoterol and budesonide (ATC code: R03AK07). The betamethasone (ATC code: H02AB01); dexamethasone (ATC code: H02AB02); methylprednisolone (ATC code: H02AB04); paramethasone (ATC code: H02AB05); prednisolone (ATC code: H02AB06); triamcinolone (ATC code: H02AB08); hydrocortisone (ATC code: H02AB09); cortisone (ATC code: H02AB10) were categories of OSs. Drug users were defined as patients who used either drug for over 30 consecutive days [22–24].

### Statistical Analyses

The distribution of categorical variables (e.g., sex, history of comorbidity, and drug used) were employed in analyzing the difference between the ACOS and non-ACOS cohorts through a chi-square test. The Student’s *t* test was employed to estimate the continuous age difference in both cohorts and to present the mean and standard deviation for the age variable. The incidence rates of PE (per 1,000 person-years) were calculated in the ACOS and non-ACOS cohorts for potential risk factors such as sex, age group (20–65 or ≥65 years old), comorbidity, and drug use. The incidence rate ratios (IRRs) and 95% CI of PE in the ACOS and non-ACOS cohorts were analyzed using a Poisson regression model. After adjustment for age, sex, comorbidity, ICSs used, and OSs used, the adjusted hazard ratios (aHRs) and 95% confidence intervals (95% CIs) of PE in the ACOS and non-ACOS cohorts were determined using Cox proportional hazard regression models. Additionally, the association between the number of outpatient visits and hospitalizations (per year) and that of PE events were estimated for the ACOS and non-ACOS cohorts by using a multivariable Cox proportional hazards model. The cumulative incidence curves for PE in both cohorts were plotted using the Kaplan–Meier method, and the curve difference was tested through a log-rank test. SAS Version 9.4 (SAS Institute, Cary, NC, USA) was employed to manage the data and for the statistical analysis. The significance level was set at *p* < 0.05 for two-sided testing.

## Results

**Fig 1:**
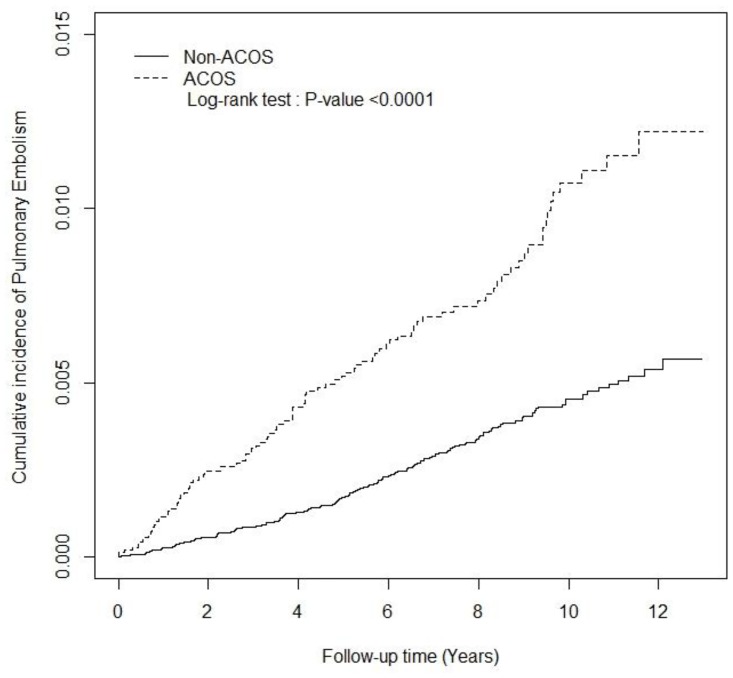
The cumulative incidence of pulmonary embolism in asthma–COPD overlap syndrome (ACOS) (dashed line) and non-ACOS cohorts (solid line).

Table 1 shows the distribution of demographic risk factors among the ACOS and non-ACOS cohorts at baseline (Table 1). There were no significant differences based on the distributions of sex and age (mean of age: 65 years old) between the cohorts. The ACOS patients had a higher prevalence of preexisting comorbidities than did the non-ACOS patients (chi square test, p < 0.0001), excluding cancer. The percentages of ICSs (27.2%) and OSs (80.2%) users were higher in ACOS cohort than in the non-ACOS cohort. During the 13-year follow-up period, the ACOS cohort had a higher cumulative incidence of PE than did the non-ACOS cohort (log-rank test, p < 0.0001; Fig 1).

**Table 1:** Comparison of demographics and history of comorbidity between ACOS and non-ACOS cohorts.

	ACOS				
	No (N = 55876)		Yes (N = 14150)		
Variable	n	%	n	%	*p*-value
**Sex**					0.27
Female	23621	42.3	5909	41.8	
Male	32255	57.7	8241	58.2	
**Age, year**					0.15
20–45	5688	10.2	1422	10.0	
45–75	35513	63.6	8898	62.9	
≥75	14675	26.3	3830	27.1	
Mean (SD)^#^	64.9	(14.2)	65.2	(14.3)	0.01
**Comorbidity**					
Atrial fibrillation	1136	2.03	514	3.63	<.0001
Hypertension	26831	48.0	8693	61.4	<.0001
Diabetes	11201	20.0	3438	24.3	<.0001
Hyperlipidemia	13008	23.3	4205	29.7	<.0001
Stroke	10306	18.4	3750	26.5	<.0001
Heart failure	2928	5.24	1705	12.0	<.0001
Fracture of lower limb	2338	4.18	767	5.42	<.0001
Cancer	1989	3.56	496	3.51	0.7548
**Medicine**					
Inhaled corticosteroids (ICSs)	1923	3.44	3855	27.2	<.0001
Oral steroids(OCs)	24401	43.7	11349	80.2	<.0001

ACOS, asthma–COPD overlap syndrome; Chi-square test;

^#^ Student’s t-test

Though the PE event is relatively rare in ACOS cohort, the overall incidence rate of PE was significantly higher in the ACOS cohort than in the non-ACOS cohort (1.02 vs. 0.42 per 1,000 person-years, respectively), and the IRR was 2.42 (95% CI: 2.29–2.55; Table 2). After adjustment for the potential risk factors, the aHR of PE was 2.08 (95% CI: 1.56–2.76) between the ACOS and non-ACOS cohorts (Table 2). The risk of PE was higher in the ACOS cohort than in the non-ACOS cohort, regardless of whether the patients were female or male. The incidence rate of PE was increased with age. Comparing the ≥65-year-old groups in each cohort, the ACOS cohort had a 1.87-fold risk of developing PE. In the comorbidity-stratified analysis and drug used-stratified analysis yielded similar results. For subjects with comorbidity, ACOS cohort had 2.00-fold the risk of PE than non-ACOS cohort (95% CI: 1.47–2.73; Table 2). The patients in the ACOS cohort who used drugs was presented 1.89-fold risk of PE than the comparison group (95% CI: 1.40–2.56; Table 2)

**Table 2:** Incidence rate and adjusted hazard ratio of pulmonary embolism between ACOS and non-ACOS cohorts stratified by sex, age, comorbidity (no/yes), and drug used (no/yes).

	ACOS							
		No			Yes		Compared to non-ACOS cohort	
Variables	Event	PY	Rate	Event	PY	Rate	IRR (95% CI)	Adjusted HR^†^ (95% CI)
**Overall**	168	399412	0.42	98	96387	1.02	2.42(2.29–2.55)***	2.08(1.56–2.76)***
**Sex**								
Female	84	174000	0.48	40	42226	0.95	1.96(1.80–2.14)***	1.63(1.06–2.50)*
Male	84	225412	0.37	58	54160	1.07	2.87(2.68–3.08)***	2.52(1.73–3.69)***
**Age, year**								
20–65	35	192150	0.18	30	47308	0.63	3.48(3.20–3.78)***	2.53(1.44–4.44)***
≥65	133	207263	0.64	68	49078	1.39	2.16(2.01–2.32)***	1.87(1.34–2.61)***
**Comorbidity**								
No	38	174432	0.22	19	26933	0.71	3.24(2.95–3.56)***	2.97(1.54–5.71)***
Yes	130	224981	0.58	79	69453	1.14	1.97(1.84–2.10)***	2.00(1.47–2.73)***
**Drug used**								
No	73	224312	0.33	18	15564	1.16	3.55(3.22–3.92)***	3.21(1.89–5.43)***
Yes	95	175100	0.54	80	80822	0.99	1.82(1.70–1.96)***	1.89(1.40–2.56)***

ACOS, asthma–COPD overlap syndrome; Drug used, including subjects with inhaled corticosteroids (ICSs) or oral steroids(OSs); PY, person-year; Rate, incidence rate (per 1,000 person-years); IRR, incidence rate ratio; Adjusted HR^†^: multiple cox model analysis including age, sex, each comorbidity, inhaled corticosteroid (ICSs), and oral steroids(OSs);

*p<0.05,

***p<0.001

The stratified analysis in each comorbidity type was showed in Table 3. Compared with non-ACOS cohort, the IRR of PE was higher in the ACOS cohort in difference comorbidity, such as atrial fibrillation (IRR: 1.87, 95% CI: 1.38–2.52), hypertension (IRR: 1.86, 95% CI: 1.73–2.01), diabetes (IRR: 2.08, 95% CI: 1.85–2.33), hyperlipidemia (IRR: 1.63, 95% CI: 1.46–1.82), stroke (IRR: 1.65, 95% CI: 1.47–1.86), fracture of lower limb (IRR: 1.86, 95% CI: 1.44–2.39), and cancer (IRR: 3.43, 95% CI: 2.60–4.52). The aHR of pulmonary embolism was obviously higher in the ACOS cohort than the non-ACOS cohort regardless of hypertension (aHR: 1.80, 95% CI: 1.27–2.56), diabetes (aHR: 2.08, 95% CI: 1.21–3.59), cancer (aHR: 3.76, 95% CI: 1.17–12.1) existence or not.

**Table 3:** Incidence rate and adjusted hazard ratio of pulmonary embolism between ACOS and non-ACOS cohorts stratified by each comorbidity types.

	ACOS							
		No			Yes		Compared to non-ACOS cohort	
Variables	Event	PY	Rate	Event	PY	Rate	IRR (95% CI)	Adjusted HR^†^ (95% CI)
**Atrial fibrillation**								
No	157	394087	0.40	89	94053	0.95	2.38(2.25–2.51)***	2.13(1.59–2.86)***
Yes	11	5325	2.07	9	2334	3.86	1.87(1.38–2.52)***	1.57(0.59–4.22)
**Hypertension**								
No	63	222429	0.28	36	40282	0.89	3.16(2.92–3.41)***	2.68(1.67–4.30)***
Yes	105	176983	0.59	62	56104	1.11	1.86(1.73–2.01)***	1.80(1.27–2.56)**
**Diabetes**								
No	128	327873	0.39	73	74866	0.98	2.50(2.35–2.66)***	2.04(1.46–2.84)***
Yes	40	71540	0.56	25	21521	1.16	2.08(1.85–2.33)***	2.08(1.21–3.59)**
**Hyperlipidemia**								
No	123	311590	0.39	74	67643	1.09	2.77(2.60–2.95)***	2.37(1.70–3.31)***
Yes	45	87822	0.51	24	28743	0.83	1.63(1.46–1.82)***	1.47(0.86–2.52)
**Stroke**								
No	120	338208	0.35	70	74789	0.94	2.64(2.48–2.80)***	2.25(1.61–3.16)***
Yes	48	61205	0.78	28	21598	1.30	1.65(1.47–1.86)***	1.62(0.96–2.71)
**Heart failure**								
No	145	385130	0.38	81	87309	0.93	2.46(2.33–2.61)***	2.25(1.65–3.06)***
Yes	23	14282	1.61	17	9077	1.87	1.16(0.96–1.41)	1.27(0.64–2.52)
**Fracture of lower limb**								
No	159	386391	0.41	93	92487	1.01	2.44(2.31–2.58)***	2.08(1.55–2.78)***
Yes	9	13021	0.69	5	3900	1.28	1.86(1.44–2.39)***	1.95(0.58–6.60)
**Cancer**								
No	161	389413	0.41	92	93884	0.98	2.37(2.24–2.51)***	2.00(1.49–2.68)***
Yes	7	9999	0.70	6	2502	2.40	3.43(2.60–4.52)***	3.76(1.17–12.1)*

ACOS, asthma–COPD overlap syndrome; PY, person-year; Rate, incidence rate (per 1,000 person-years); IRR, incidence rate ratio; Adjusted HR^†^: multiple cox model analysis including age, sex, each comorbidity, inhaled corticosteroid (ICSs), and oral steroids(OSs);

*p<0.05,

**p<0.01,

***p<0.001

Table 4 presents the risk of PE in the ACOS patients using ICSs or OSs. Compared with the non-ACOS cohort, the risk of PE was presented 1.97-fold (95% CI: 1.29–3.01) in ACOS patients who received ICSs treatment. The aHR of PE decreased from 2.91 (95% CI: 1.82–4.63) to 1.97 (95% CI: 1.46–2.65) in the ACOS patients using OSs.

**Table 4:** Adjusted hazard ratio of pulmonary embolism found in the follow-up period associated with ACOS and prescriptions of ICSs and OSs.

Variables	N	Event	Rate	IRR (95% CI)	Adjusted HR^†^ (95% CI)
**Non-ACOS cohort**	55876	168	0.42	1.00	1.00
**ACOS cohort**					
Without Inhaled corticosteroids (ICSs)	10295	71	1.05	2.50(2.36–2.66)***	2.22(1.66–2.98)***
With Inhaled corticosteroids (ICSs)	3855	27	0.93	2.21(2.03–2.42)***	1.97(1.29–3.01)**
**Non-ACOS cohort**	55876	168	0.42	1.00	1.00
**ACOS cohort**					
Without oral steroids(OSs)	2801	20	1.11	2.64(2.39–2.92)***	2.91(1.82–4.63)***
With oral steroids (OSs)	11349	78	0.99	2.37(2.23–2.51)***	1.97(1.46–2.65)***

ACOS, asthma–COPD overlap syndrome; Rate, incidence rate (per 1,000 person-years); IRR, incidence rate ratio; Adjusted HR^†^: multiple cox model analysis including age, sex, each comorbidity, inhaled corticosteroid (ICSs), and oral steroid(OSs);

**p<0.01,

***p<0.001

Compared with the non-ACOS cohort, the risk of developing PE was more notably affected by the number of outpatient visits and hospitalizations necessitated in the ACOS cohort, ranging from 2.32 (95% CI: 1.54–3.52) for those having 3–9 visits to 4.20 (95% CI: 2.74–6.44) for those having >9 visits (*p* < 0.0001; Table 5).

**Table 5:** The adjusted hazard ratio of pulmonary embolism associated with number of outpatient visits and hospitalizations per year due to COPD or asthma exacerbation.

Variables	N	Event	Rate	IRR (95% CI)	Adjusted HR^†^ (95% CI)
**Non-ACOS cohort**	55876	168	0.42	1.00	1.00
**ACOS cohort**					
**Number of outpatient visits and hospitalizations per year**					
≤ 3	7014	32	0.59	1.41(1.30–1.53)***	1.42(0.96–2.10)
3–9	4052	32	1.17	2.79(2.57–3.02)***	2.32(1.54–3.52)***
>9	3084	34	2.27	5.40(4.98–5.84)***	4.20(2.74–6.44)***
*p-value for trend*				<.0001	<.0001

ACOS, asthma–COPD overlap syndrome; Rate, incidence rate (per 1,000 person-years); IRR, incidence rate ratio; Adjusted HR^†^: multiple cox model analysis including age, sex, each comorbidity, inhaled corticosteroid (ICSs), and oral steroid(OSs);

***p<0.001

## Discussion

The eosinophilic [18] and neutrophilic [22] inflammation [28] of the airway with pulmonary artery inflammation [12,13] might be the predisposing factors of PE [18] in previous study [15,16]. Meanwhile, the higher level of neutrophil cells with hemostatic markers (e.g., the ETP and vWF) of a prothrombotic state [14] have the effect on the hypercoagulation. Combined these factors, our study show that the ACOS have an impact on risk of the PE, especially in the patients receiving the high frequency of the healthcare utility [29]; even in the young adults or [30] people without comorbidities. In addition, complications such as hypertension [24], diabetes and cancer [31] were associated with the atherosclerosis in venous and arterial disease [32,33], which might play a role in the risk of PE [4,5] among the ACOS patients also.

These two phenotypes are found in the ACOS phenotypes such as Th2 high eosinophil predominant, Th2 low neutrophil predominant [19]. When the eosinophil predominant phenotype responds favorably to steroids [34], the level of hypoxeamia may be less [35], thus having the less risk of the hypercoagulation [36]. Similarly, in Wedzicha JA et al. report, the higher the blood eosinophil count, the greater the AE reduction response to ICSs. This finding might imply that the blood eosinophil count measured at a COPD with AE can direct the use of OSs at AE [28]. Moreover, according to Chen et al., high-dose ICSs use in patients with COPD improved their lung function status, decreased the frequency of exacerbations, and improved symptom relief, thus having the less risk of hypoxieamia [37]. These previous findings may explain why the ACOS cohort using steroids (aHR = 1.97 for ICSs and OSs) had a lower risk of PE than those not using steroids (aHR = 2.22 for ICSs; 2.91 for OSs) in the current study. Furthermore, Sneeboer et al. found that patients using OSs (odds ratio 0.5; *p*<0.01) or ICSs (odd ratio 0.5; *p* = 0.02) used these steroids >6 months prior to the PE event; this protective effect of PE in line with our findings [38].

Anti-inflammatory drugs, particularly ICSs [33], are the mainstay of treatment for asthma or AE of COPD cohort [33]. ACOS is also a systemic inflammation disease, for which ICSs are usually applied in treatment [39] The higher frequency of PE among ACOS patients receiving more medical services accords with this finding.

Steroids-induced hypercoagulability has reported for several years; however it remains controversial whether the use of steroids or the underlying disease itself contributes to the hypercoagulable state [15]. The present study highlights the role of PE in ACOS cohort; the patients who used steroids may have had a lower risk of PE than those who did not. Steroids use may reduce hypoxeamia [37,39], thus patients receiving steroids may have the less risk of hypercoagulation [28]. However, it may also induce the prothrombotic state, irrespective of the dose, the risk of PE may be higher in these patients with steroids use, especially during the first month of exposure [40]. Determining the appropriate steroids dosage and a means of shorting the duration of steroid use for the ACOS cohort with incidental PE, while avoiding PE, warrants randomized control study.

In the Kumbhare S et al. report [31], they compared the ACOS cohort with the pure COPD cohort and the pure asthma cohort. The ACOS cohort was more likely to have at least one co-morbidity, more hospitalization or emergency department visits, less exercise and more disability compared to the pure COPD cohort. Similarly, the comorbidities (e.g, the diabetes [24], hypertension, cancer [24]), acute respiratory event [24] (less exercise) and higher frequency of medical services (disability) are more associated with the ACOS cohort in our study. These factors were predisposing factors of the PE. In van Boven JF et al. study [41], they found that allergic rhinitis, anxiety, osteoporosis and gastroesophageal reflux disease are more related to the ACOS cohort than the pure COPD cohort, while ischemia heart disease (IHD) and chronic kidney disease were less associated. Regarding the impact on hospitalization risk in this previous study, the comorbidities such as lung cancer [9] and cor pulmonale [8] had relatively the strongest association with 1-year all-cause hospitalization risk in the ACOS cohort [41]. Meanwhile, these two factors [8,9] are associated with the PE also [42]. In their study did not include PE, and that would warrant further investigation, likewise for the differences between risk for PE in ACOS cohort and pure asthma cohort alone.

The management in an individual patient with PE requires clinical assessment of risks and benefits and also depends on local availability of therapeutic interventions [43]. Incidental and isolated subsegmental PE should generally be managed in the same manner as symptomatic and non-subsegmental PE [43]. This hypothesis, however, should be assessed in prospective clinical trials [44]. By contrast, patients diagnosed with acute symptomatic PE, concomitant DVT was significantly associated with an increased risk of death within 30 days of PE diagnosis [40,45]. In addition, long-term Outcomes after PE (ELOPE) Study indicate that almost half of PE patients can be considered to have a "post-PE syndrome" characterized by exercise limitation at 1 year, which influences their quality of life (QOL) and degree of dyspnea [46]. The higher frequency of medical services [31] and the post-PE syndrome aggravated the disability [29]. Therefore, this study should alert the clinicians to be aware of the incident PE in the ACOS cohort, even in young adults or people without comorbidities.

### Strengths

This is the first cohort study discussing the relationship between PE and ACOS based on the general population. PE-associated comorbidities and medicine (ICSs and OSs) were considered in this study. Multidisciplinary services for care of ACOS [24] and PE patients are well established in Taiwan [25]. Courses of ACOS and PE treatment can be followed up by public nurses and physicians. Furthermore, regular use of drugs such as ICSs can be monitored under these strict services. Meanwhile, our study cohort enrolled the patients aged >20 years <40 years [23] for avoid missing the young adults with ACOS.

### Limitations

Several limitations were encountered in this study. The first limitation is the definition of ACOS in this paper and this is a very contentious area already, the ACOS cohort in this study is based on the COPD-subset. Second, even in ACOS, the incidence of PE is 1.02 in 1000 person years. Third, this study does not mention if the PE are incidental findings or not. The management of incidental PE is uncertain and patients with ACOS are at high risk of over-diagnosis of asymptomatic PE. Fourth, some of the important clinical variables/risk factors that have been associated with PE were not included such smoking, body mass index, pregnancy, use of oral anticonceptives (in women), anticoagulant drugs (aspirin, warfarin) and genetics. Fifth, we did not analyze the effect of bronchodilators or erythromycin on PE. Sixth, the ETP and vWF levels are unavailable in the NHIRD. Finally, clinical parameters related to asthma/COPD such as lung function parameters (e.g. forced expiratory volume in 1 second %, DLco %, 6MWT) are unavailable at NHIRD also. These confounding factors warrant investigation.

## Conclusions

ACOS is associated with increased risk of PE, particularly patients with a high frequency of AE—even in young adults or people without comorbidities.

## Supporting Information

S1 Checklist STROBE Statement—checklist of items that should be included in reports of observational studies. (DOC)

## Abbreviations

aHR: adjusted hazard ratio
CI: confidence interval
NHIRD: National Health Insurance Research Database
NHI: National Health Insurance
NHRI: National Health Research Institutes
LHID 2000: Longitudinal Health Insurance Database 2000
ACOS: asthma–chronic obstructive overlap syndrome
PE: pulmonary embolism
